# Dynamics analysis of a delayed virus model with two different transmission methods and treatments

**DOI:** 10.1186/s13662-019-2438-0

**Published:** 2020-01-06

**Authors:** Tongqian Zhang, Junling Wang, Yuqing Li, Zhichao Jiang, Xiaofeng Han

**Affiliations:** 1 College of Mathematics and Systems Science, Shandong University of Science and Technology, Qingdao, P.R. China; 2 Fundamental Science Department, North China Institute of Aerospace Engineering, Langfang, P.R. China

**Keywords:** 39A28, 39A30, 92B05, Cell-to-cell transmission, Delayed virus model, Treatment, Hopf bifurcation

## Abstract

In this paper, a delayed virus model with two different transmission methods and treatments is investigated. This model is a time-delayed version of the model in (Zhang et al. in Comput. Math. Methods Med. 2015:758362, 2015). We show that the virus-free equilibrium is locally asymptotically stable if the basic reproduction number is smaller than one, and by regarding the time delay as a bifurcation parameter, the existence of local Hopf bifurcation is investigated. The results show that time delay can change the stability of the endemic equilibrium. Finally, we give some numerical simulations to illustrate the theoretical findings.

## Introduction and model formulation

Infectious diseases are still important diseases that endanger human health [2]. Not only do some ancient infectious disease pathogens continue to mutate and change, but new pathogens are also emerging, bringing many difficulties for us to discover, diagnose, and prevent infectious diseases. Studies have shown that many diseases are caused by viruses. Of more than 4000 viruses discovered so far, more than 100 can directly threaten human health and life. For example, rabies, a zoonotic disease caused by rabies virus, can cause severe encephalitis. Because the virus invades the central nervous system, if the treatment is not taken in time, the mortality rate is almost 100%. Another example is the Ebola virus, which causes Ebola hemorrhagic fever with a mortality rate from 50% to 90%. In addition, recent research has shown that several viruses have been found to link with cancer in humans, even that can push the cell toward becoming cancerous, such as human papilloma viruses (HPVs) which are considered to be the biggest factor that causes various cancers such as cervical cancer [3–5], anal cancer [6, 7], and oropharyngeal cancers [8]. The sudden outbreak of the SARS virus and the Ebola virus in the past 20 years has given us a major warning that public health issues are no longer just health issues, but also an important part of national security and urban security systems.

Using mathematical models to help discover the mechanism of viral transmission to predict the development of infectious diseases has become the mainstream method for controlling and preventing infectious diseases [9–12]. Therefore, for over a century, lots of mathematical models have been established to explain the evolution of the free virus in a body, and mathematical analysis was implemented to explore the threshold associated with eradication and persistence of the virus; for example, [13–16] studied the global dynamic behavior of HIV models, [17–24] analysed the global dynamics of HBV models [25–28]. A general class of models describing the process of virus invading the target cells and release of the virus due to the infected cell apoptosis has been established and analyzed by Perelson et al. [29, 30] and Nelson et al. [31] as follows:

1 $$ \textstyle\begin{cases} {\frac{dx}{dt} =\varLambda -dx-\alpha v x,} \\ {\frac{dy}{dt} =\alpha v x-ay,} \\ {\frac{dv}{dt} =ky-uv,} \end{cases} $$

where *x*, *y*, and *v* represent the concentrations of uninfected target cells, infected cells, and virus, respectively. *Λ* and *d* are the generation rate and mortality rate of uninfected target cells respectively, *α* is the infection rate, *a* is the mortality rate of infected cells, *k* and *u* are the generation rate and mortality rate of free virus respectively.

However, the above model only considers that free viruses can infect uninfected cells by direct contact with them. Recent studies have shown that virus can be transmitted directly from cell to cell by virological synapses, i.e., cell-to-cell transmission [32–39]. Spouge et al. [40] built a model to characterize this phenomenon:

2 $$ \textstyle\begin{cases} {\frac{dC}{dt} =r_{C}C (1-\frac{C+I+\gamma M}{C_{m}} )-k _{I}IC,} \\ {\frac{dI}{dt} = k_{I}IC-\mu _{I}I,} \\ {\frac{dM}{dt} = \mu _{C}C+\mu _{I}I,} \end{cases} $$

where *C*, *I*, *M* represent the concentrations of uninfected cells, infected cells, and dead cells, respectively. $k_{I}$ is the rate constant for cell-to-cell spread, $r_{C}$ is the reproductive rate of uninfected cell. $\mu _{I}$, $\mu _{C}$ are the rate constants at which uninfected or infected cells die respectively, and the term $k_{I}IC$ represents the cell-to-cell transmission. However, research [41] shows that there is a delay between the time an uninfected cell becomes infected and when it begins to infect other uninfected cells, then Culshaw et al. [42] improved the model by introducing a distributed delay into model (2) and got the following model:

$$ \textstyle\begin{cases} {\frac{dC}{dt} = r_{C}C(t) (1-\frac{C(t)+I(t)}{C_{M}} )-k _{I}C(t)I(t),} \\ {\frac{dI}{dt} = k'_{I}\int ^{t}_{-\infty }C(u)I(u)F(t-u)\,du-\mu _{I}I(t),} \end{cases} $$

where $F(u)$ is the delay kernel.

Lai et al. [43] proposed a model containing two different types of infection as follows:

$$ \textstyle\begin{cases} {\frac{dT}{dt} = rT(t) (1-\frac{T(t)+\alpha T^{*}(t)}{T_{M}} )- \beta _{1} T(t)V(t)-\beta _{2} T(t)T^{*}(t),} \\ {\frac{dT^{*}}{dt} = \beta _{1} T(t)V(t)+\beta _{2} T(t)T^{*}(t)-d_{T _{*}}T^{*}(t),} \\ {\frac{dV}{dt} = kT^{*}(t)-d_{V}V(t),} \end{cases} $$

where *T*, $T^{*}$, and *V* are the concentrations of uninfected cells, infected cells, and free virus, respectively. $\beta _{1}$ is the infection rate of cell-free virus transmission and $\beta _{2}$ is the infection rate of cell-to-cell transmission. For more details on parameters, please see [43]. The authors proved that a Hopf bifurcation can occur under certain conditions. Recently, Zhang et al. [1] proposed an ordinary differential equations virus model with both two different types of infection and cure rate as follows:

3 $$ \textstyle\begin{cases} {\frac{dx}{dt} = \pi -dx-(\beta y+\alpha v)x+\rho y,} \\ {\frac{dy}{dt} = (\beta y+\alpha v)x-(a+\rho )y,} \\ {\frac{dv}{dt} = ky-uv,} \end{cases} $$

where *x*, *y*, and *v* represent the concentrations of uninfected cells, infected cells, and free virus respectively. *π* is the regeneration rate of uninfected cells, *d*, *a*, *u* are the death rates of three kinds of cells. *ρ* represents the cure rate, *ky* is the rate at which infected cells produce free viruses. By constructing suitable Lyapunov function, the authors proved that the equilibria are globally asymptotically stable under some conditions. However, the authors did not consider the time delay in model (3). In order to understand whether the introduction of time delay or not will change the stability of the equilibria, then motivated by the works [42, 43] and based on [1], we further consider model (4) by introducing a discrete delay into model (3) as follows:

4 $$ \textstyle\begin{cases} {\frac{dx}{dt} = \pi -dx(t)-(\beta y(t-\tau )+\alpha v(t-\tau ))x(t- \tau )+\rho y(t),} \\ {\frac{dy}{dt}=(\beta y(t-\tau )+\alpha v(t-\tau ))x(t-\tau )-(a+ \rho )y(t),} \\ {\frac{dv}{dt}=ky(t)-uv(t),} \end{cases} $$

where *τ* is time delay. $\beta xy$ is the term of cell-to-cell transmission. $\alpha vx$ represents cell-free virus transmission. For more details on parameters, please see [1]. Considering the biological meanings, we analyze model (4) in region $A = \{(x, y, v)\in R^{3}_{+}\vert 0\leq x + y \leq {\pi }/{\lambda }, v \geq 0\}$, where $\lambda =\min \{a,d\}$.

The paper is organized as follows. Firstly, we summarize some basic results about model (4) in Sect. 2. The local stability of the free equilibrium and Hopf bifurcation of the system are discussed in Sect. 3. We give the properties of Hopf bifurcation in Sect. 4. Finally, we perform some numerical simulations to verify the results in Sect. 5.

## Some basic results

From [1], we can conclude some basic results and summarize them in the following theorem in this section.

### Theorem 2.1

(i) *Model* (3) *or* (4) *always has a virus*-*free equilibrium* $E_{0} =(x_{0},0,0)$, *where* $x_{0}={\pi }/{d}$.
(ii) *If* $\mathscr{R}>1 $, *model* (3) *or* (4) *has a unique endemic equilibrium* $E_{1}(x^{*},y^{*},v^{*}) $, *where*
  $$ x^{*}=\frac{\pi }{d \mathscr{R}}, \quad\quad y^{*}=\frac{\pi }{a} \biggl(1- \frac{1}{ \mathscr{R}} \biggr), \quad\quad v^{*} =\frac{k }{u}y^{*}, $$
  *and*
  $$ \mathscr{R}=\frac{\pi (\alpha k+\beta u)}{du(a+\rho )} $$
  *is the basic reproduction number*.

## Local stability of the free equilibrium and Hopf bifurcation

### Theorem 3.1

*For model* (4), *if* $\mathscr{R}<1$, $E_{0}$*is locally stable*, *and if* $\mathscr{R}>1$, *then* $E_{0}$*is unstable*.

### Proof

Letting $X=x-x_{0}$, $Y=y$, $V=v$ in (4) yields

5 $$ \textstyle\begin{cases} {\frac{dX}{dt} =\pi -d(X(t)+x_{0})-(\beta Y(t-\tau )+\alpha V(t- \tau ))(X(t-\tau )+x_{0})+\rho Y(t),} \\ {\frac{dY}{dt} =(\beta Y(t-\tau )+\alpha V(t-\tau ))(X(t-\tau )+x_{0})-(a+ \rho ) Y(t),} \\ {\frac{dV}{dt} = k Y(t)- u V(t)}. \end{cases} $$

Then linearization at the original results in a characteristic equation is as follows:

6 $$ (\lambda +d)\bigl[\lambda ^{2}+(u +a+\rho )\lambda +(a+ \rho )u+(-\beta x _{0} \lambda -\beta ux_{0}- \alpha k x_{0})e^{-\lambda \tau }\bigr]=0. $$

Clearly, it has a root $\lambda =-d<0$. Thus we only need to analyze the distribution of roots, which determines the stability of solution of system (5) of the equation

7 $$ P_{1}(\lambda )+{P_{2}}(\lambda )e^{-\lambda \tau }=0, $$

where

$$ P_{1}(\lambda )=\lambda ^{2}+A\lambda +B, \quad\quad P_{2}( \lambda )=C\lambda +D $$

and

$$ A=a+\rho + u>0,\quad\quad B= (a+\rho )u>0, \quad\quad C=-\beta x_{0}< 0,\quad\quad D=-(\beta u+\alpha k)x_{0}< 0. $$

When $\tau =0$, (7) reduces to

8 $$ \lambda ^{2}+(A+C)\lambda +B+D=0. $$

Since $\mathscr{R}=\frac{(\alpha k+\beta u)x_{0}}{(a+\rho )u}<1$ implies $A+C>0$ and $B+D>0$, we know the two roots of (8) always have a negative real part. Next, we assume that equation (7) with $\tau \neq 0$ has two pure imaginary roots $\pm i\varpi$ ($\varpi > 0$), which implies that the equation

9 $$ F(\varpi )=\varpi ^{4}+\bigl(A^{2}-C^{2}-2B \bigr)\varpi ^{2}+B^{2}-D^{2}=0 $$

has at least one positive solution.

Obviously, $B^{2}-D^{2}>0$, then we can see that

$$\begin{aligned} A^{2}-C^{2}-2B &=(a+\rho + u)^{2}-(\beta x_{0})^{2}-2(a+\rho )u \\ &=(a+\rho )^{2}+ u^{2}-(\beta x_{0})^{2} \\ &>0, \end{aligned}$$

where $a+\rho >\beta x_{0}$ is used. Then we can conclude that when $\mathscr{R}<1$, equation (9) has no positive real root, which leads to that equation (7) does not have a pure imaginary root. Thus, all the roots of (7) always have negative real parts. Therefore, $E_{0}$ is locally stable. When $\mathscr{R}>1$, it is easy to see $B+D<0$, then equation (7) has at least one positive root, thus $E_{0}$ is unstable. This proof is completed. □

Next we discuss the existence of Hopf bifurcation. For this purpose, we let $X=x-x^{*}$, $Y=y-y^{*}$, $V=v-v^{*}$, to shift the equilibrium to the original. Then linearization at the original results in a characteristic equation is as follows:

10 $$ \Delta (\lambda ,\tau )=\lambda ^{3}+b_{2} \lambda ^{2}+b_{1}\lambda +b _{0}+ \bigl(c_{2}\lambda ^{2}+c_{1}\lambda +c_{0}\bigr)e^{-\lambda \tau }=0, $$

where

$$\begin{aligned}& b_{0} =d (a+\rho ) u, \\& b_{1} =d (a+\rho +u) +(a+\rho )u, \\& b_{2} =d +a+\rho +u , \\& c_{0} =au\bigl(\beta y^{*} + \alpha v^{*} \bigr)-d\bigl(\beta ux^{*}+\alpha kx^{*}\bigr), \\& c_{1} =(u+a) \bigl(\beta y^{*}+\alpha v^{*} \bigr)-x^{*}(\beta u+\alpha k)-d \beta x^{*}, \\& c_{2} =\beta y^{*} + \alpha v^{*}-\beta x^{*}. \end{aligned}$$

When $\tau = 0$, (10) reduces to

$$ {\Delta (\lambda ,0)=f(\lambda )}=\lambda ^{3}+a_{2}\lambda ^{2}+a_{1} \lambda +a_{0}=0, $$

where

$$\begin{aligned}& \begin{aligned} a_{2} &=b_{2}+c_{2} \\ &=d +a+\rho +u+\beta y^{*} + \alpha v^{*}-\beta x^{*} \\ &=d +u+\beta y^{*} + \alpha v^{*}+ \alpha k/ux^{*}>0, \end{aligned} \\& \begin{aligned} a_{1} &=b_{1}+c_{1} \\ &=d (a+\rho +u) +(a+\rho )u+(u+a) \bigl(\beta y^{*}+\alpha v^{*}\bigr)-x^{*}( \beta u+\alpha k)-d\beta x^{*} \\ &=d u +(u+a) \bigl(\beta y^{*}+\alpha v^{*}\bigr)+ d\alpha k/u x^{*}>0, \end{aligned} \\& \begin{aligned} a_{0} &=b_{0}+c_{0} \\ &=au\bigl(\beta y^{*}+\alpha v^{*}\bigr)>0, \end{aligned} \end{aligned}$$

here $(a+\rho )u=(\beta u+\alpha k)x^{*}$ is used. And

$$\begin{aligned} a_{2}a_{1}-a_{0}&= \bigl(d +u+\beta y^{*} + \alpha v^{*}+ \alpha k/ux^{*}\bigr) \bigl(d u +(u+a) \bigl(\beta y^{*}+\alpha v^{*}\bigr)+ \alpha k/u x^{*}\bigr) \\ &\quad{} -au\bigl(\beta y^{*}+\alpha v^{*}\bigr) \\ &= \bigl(d+\beta y^{*} + \alpha v^{*}+ \alpha k/ux^{*}\bigr) \bigl(d u +(u+a) \bigl(\beta y ^{*}+\alpha v^{*}\bigr)+ \alpha k/u x^{*}\bigr) \\ &\quad{} +u \bigl(d u +u\bigl(\beta y^{*}+\alpha v^{*} \bigr)+ \alpha k/u x^{*}\bigr) \\ &>0. \end{aligned}$$

Then all the roots of $f(\lambda )=0$ have negative real parts by using the Routh–Hurwitz criterion [44], which implies the local asymptotic stability of $E^{*}$ for $\tau =0$.

For $\tau >0$, let *iω* ($\omega >0$) be the root of (10), we get

11 $$ \textstyle\begin{cases} \sin \omega \tau =\frac{c_{2} \omega ^{5}+(b_{2}c_{1}-b_{1}c_{2}-c _{0})\omega ^{3}+(b_{1}c_{0}-b_{0}c_{1})\omega }{(c_{2}\omega ^{2}-c _{0})^{2}+c_{1}^{2}\omega ^{2}}=P(\omega ), \\ \cos \omega \tau =\frac{(c_{1}-b_{2}c_{2})\omega ^{4}+(b_{0}c_{2}+b _{2}c_{0}-b_{1}c_{1})\omega ^{2}-b_{0}c_{0}}{(c_{2}\omega ^{2}-c_{0})^{2}+c _{1}^{2}w^{2}}=Q(\omega ). \end{cases} $$

By setting $\mu = \omega ^{2}$, we get

12 $$ G(\mu )=\mu ^{3}+d_{2}\mu ^{2}+d_{1}\mu +d_{0}=0, $$

where

$$\begin{aligned}& d_{0} =b_{0}^{2}-c_{0}^{2}, \\& d_{1} =b_{1}^{2}+2c_{0}c_{2}-2b_{0}b_{2}-c_{1}^{2}, \\& d_{2} =b_{2}^{2}-2b_{1}-c_{2}^{2}. \end{aligned}$$

By the method in [45], we get the following lemmas.

### Lemma 3.1

(i) *If conditions* $b_{0}>c_{0}$*and* $d_{2}^{2}-3d_{1}\leq 0$*hold*, *then* (12) *has no positive root*.
(ii) *If conditions* $b_{0}>c_{0}$, $(H_{1}) d_{2}^{2}-3d_{1}>0$, $z_{1} ^{*}>0$, *and* $G(z_{1}^{*})<0$*hold*, *then* (12) *has two positive roots*, *where* $z_{1}^{*}=\frac{-d_{2}+\sqrt{d_{2}^{2}-3d _{1}}}{3}$.
(iii) *If condition* $b_{0}< c_{0}$*holds*, *then* (12) *has at least one positive root*.

If $b_{0}>c_{0}$ and $(H_{1})$ hold, suppose $z_{1}< z_{2}$, then $\frac{dG}{dz_{1}}<0$ and $\frac{dG}{dz_{2}}>0$. Substituting $\omega _{k}=\sqrt{z_{k}}$ ($k=1,2$) into (11), we have

13 $$ \tau _{k}^{(j)}= \textstyle\begin{cases} \frac{1}{\omega _{k}}[\arccos (Q(\omega _{k}))+2j\pi ], &P(\omega _{k})\geq 0, \\ \frac{1}{\omega _{k}}[2\pi -\arccos (Q(\omega _{k}))+2j\pi ], &P(\omega _{k})< 0, \end{cases}\displaystyle \quad j=0, 1, 2,\ldots . $$

### Lemma 3.2

*If* (*H*1) *holds*, *then* $\frac{d\alpha }{d\tau _{1}}<0$*and* $\frac{d\alpha }{d\tau _{2}}>0$, *where* $\lambda (\tau )=\alpha (\tau )+i\omega ( \tau )$*is the root of* (10) *and* $\alpha (\tau _{k}^{(j)})=0$, $\omega (\tau _{k}^{(j)})=\omega _{k}$ ($k=1,2$; $j=0,1,2,\ldots $).

### Proof

Substituting $\lambda (\tau )$ into (10), we get

$$ \biggl[\frac{d\alpha }{d\tau _{k}^{(j)}} \biggr]^{-1}=\frac{3{\omega _{0}}^{4}+2d_{2}{\omega _{0}}^{2}+d_{1}}{c_{1}{\omega _{0}}^{2}+(c_{0}-c _{2}{\omega _{0}}^{2})^{2}{\omega _{0}}^{2}}= \frac{\frac{dG}{dZ_{k}}}{c _{1}{\omega _{0}}^{2}+(c_{0}-c_{2}{\omega _{0}}^{2})^{2}{\omega _{0}} ^{2}}. $$

Obviously, $\operatorname{Sign}\frac{d\alpha }{d\tau _{k}^{(j)}}=\operatorname{Sign}\frac{dG}{dz _{k}}$. Since $\frac{dG}{dz_{1}}<0$ and $\frac{dG}{dz_{2}}>0$, then we have $\frac{d\alpha }{d\tau _{1}}<0$ and $\frac{d\alpha }{d\tau _{2}}>0$. The proof is completed. □

About the existence of Hopf bifurcation, we have the following theorem.

### Theorem 3.2

*If* $\mathscr{R}>1$, $b_{0}>c_{0}$, *and* $(H_{1})$*hold*, *then system* (4) *undergoes a Hopf bifurcation at* $E^{*}$*when* $\tau =\tau _{k}^{(j)}$ ($k=1,2$; $j=0,1,2,\ldots $). *Furthermore*, $E^{*}$*is stable when* $\tau \in [0,\tau _{0})$*and unstable when* $\tau >\tau _{0}$. $\tau _{0}$*is the Hopf bifurcation value*.

## Property of Hopf bifurcation at $E^{*}$

From Theorem 3.2, we got the sufficient conditions for the Hopf bifurcation to appear. We assume that when $\tau =\tau _{0}$, system (4) produces a Hopf bifurcation at $E^{*}$. Next by the normal form theory and the center manifold [46], we try to establish the explicit formula determining the directions, stability, and period of periodic solutions bifurcating from $E^{*}$ at $\tau =\tau _{0}$ and $\omega (\tau _{0})=\omega _{0}$.

Let $\omega _{0}=\omega (t_{0})$, $\tau =\tau _{0}+\varsigma $, $\varsigma \in \mathbb{R}$, then $\varsigma =0$ is the Hopf bifurcation value of model (4). Let $w_{1}(t)=x-x^{*}$, $w_{2}(t)=y-y^{*}$, $w_{3}(t)=v-v ^{*}$, then (4) becomes

14 $$\begin{aligned} \textstyle\begin{cases} {\frac{dw_{1}}{dt}}= -dw_{1}(t)+\rho w_{2}(t)-(\beta y^{*}+\alpha v ^{*})w_{1}(t-\tau )-\beta x^{*}w_{2}(t-\tau )-\alpha x^{*}w_{3}(t- \tau ) \\ \hphantom{\frac{dw_{1}}{dt}}\quad{} -\beta w_{1}(t-\tau )w_{2}(t-\tau )t-\alpha w_{1}(t-\tau )w_{3}(t- \tau ), \\ {\frac{dw_{2}}{dt}}= -(a+\rho )w_{2}(t)+(\beta y^{*}+\alpha v^{*})w _{1}(t-\tau )+\beta x^{*}w_{2}(t-\tau )+\alpha x^{*}w_{3}(t-\tau ) \\ \hphantom{\frac{dw_{2}}{dt}}\quad{} +\beta w_{1}(t-\tau )w_{2}(t-\tau )+\alpha w_{1}(t-\tau )w_{3}(t- \tau ), \\ {\frac{dw_{3}}{dt}}= kw_{2}(t)-uw_{3}(t). \end{cases}\displaystyle \end{aligned}$$

Let $C = C([-\tau ,0],R^{3})$, we have

15 $$ \dot{w}_{t}=L_{\varsigma }w_{t}+F(\varsigma ,w_{t}), $$

where $w_{t}(\theta )=w(t+\theta )\in C$ and $L_{\varsigma }$ is given by

$$ L_{\varsigma } \varphi =B_{1}\varphi (0) +B_{2}\varphi (- \tau _{0}), $$

where $\varphi =(\varphi _{1}, \varphi _{2}, \varphi _{3})^{T}$,

$$\begin{aligned}& B_{1}= \begin{pmatrix} -d & \rho & 0 \\ 0 & -(a+\rho ) & 0 \\ 0 & k & -u \end{pmatrix}, \quad\quad B_{2}= \begin{pmatrix} -(\beta y^{*}+\alpha v^{*}) & -\beta x^{*} & -\alpha x^{*} \\ \beta y^{*}+\alpha v^{*} & \beta x^{*} & \alpha x^{*} \\ 0 & 0 & 0 \end{pmatrix}, \\& F(\varsigma ,w_{t})= \begin{pmatrix} -\beta w_{1}(t-\tau )w_{2}(t-\tau )-\alpha w_{1}(t-\tau )w_{3}(t- \tau ) \\ \beta w_{1}(t-\tau )w_{2}(t-\tau )+\alpha w_{1}(t-\tau )w_{3}(t- \tau ) \\ 0 \end{pmatrix}. \end{aligned}$$

By using the Riesz representation theorem, we have a function $\zeta (\theta ,\varsigma )$ such that, for $\varphi \in C$,

$$ L_{\varsigma }\varphi = \int _{-\tau _{0}}^{0} \,d\zeta (\theta ,\varsigma )\varphi (\theta ), $$

where $\zeta (\theta ,\varsigma )$ is a bounded variation function for $[-\tau _{0},0]$. And we can choose

$$ \zeta (\theta ,\varsigma )=B_{1}\delta (\theta )-B_{2} \delta (\theta +\tau ), $$

where

$$ \delta (\theta )= \textstyle\begin{cases} 1,&\theta =0, \\ 0,&\theta \neq 0. \end{cases} $$

For $\varphi \in C^{1}=C([0,\tau ],R^{3*})$, let us define

$$ H(\varsigma )\varphi = \textstyle\begin{cases} \dot{\varphi }(\theta ), & \theta \in (-\tau _{0},0), \\ \int _{-\tau _{0}}^{0} \,d\zeta (s,\varsigma )\varphi (s), & \theta =0, \end{cases} $$

and

$$ R(\varsigma )\varphi = \textstyle\begin{cases} 0, & \theta \in [-\tau ,0), \\ F(\varsigma ,\varphi ), & \theta =0 \end{cases} $$

is the nonlinear part of the right-hand side of system (15), where

$$\begin{aligned} F(\varsigma ,\varphi )&= \begin{pmatrix} -\beta \varphi _{1}(-\tau )\varphi _{2}(-\tau )-\alpha \varphi _{1}(- \tau )\varphi _{3}(-\tau ) \\ \beta \varphi _{1}(-\tau )\varphi _{2}(-\tau )+\alpha \varphi _{1}(- \tau )\varphi _{3}(-\tau ) \\ 0 \end{pmatrix}. \end{aligned}$$

For $\psi \in C^{1}([0,\tau _{0}],R^{3\ast })$ and $\varphi \in C([- \tau _{0},0],R^{3})$, define

$$ H^{\ast }\psi (s)= \textstyle\begin{cases} -\dot{\psi }(s), & s\in (0,\tau _{0}], \\ \int _{-\tau _{0}}^{0}\,d\zeta ^{T}(t,0)\psi (-t), & s=0, \end{cases} $$

and

$$ \langle \psi ,\varphi \rangle = \bar{\psi }(0)\varphi (0)- \int _{-\tau _{0}}^{0} \int _{\xi =0}^{\theta } \bar{\psi }^{T}(\xi - \theta )\,d\zeta (\theta )\varphi (\xi )\,d\xi , $$

where $\zeta (\theta )=\zeta (\theta ,0)$. We have that $H^{\ast }$ and $H=H(0) $ are adjoint operators. Then $\pm i\omega _{0}$ are eigenvalues of $H(0)$ when $\tau =\tau _{0}$. Thus they are also eigenvalues of $H^{\ast }$. Also, we can get $q(\theta )=(1,q_{2},q_{3})e^{i\omega _{0}\theta }$ and $q^{\ast }(s)=\bar{D}(1,q_{2}^{*},q_{3}^{*})e^{i \omega _{0}s}$, which are the eigenvectors of *H* and $H^{\ast }$ corresponding to $i\omega _{0}$ and $-i\omega _{0}$, respectively, and

$$\begin{aligned}& h_{2}= -\frac{i\omega _{0}+d}{i\omega _{0}+a}, \\& h_{3}= \frac{ (i\omega _{0}+a+\rho -\beta x^{*} e^{-i\omega _{0} \tau _{0}} )h_{2}- (\beta y^{*}+\alpha v^{*} )e^{-i\omega _{0} \tau _{0}}}{\alpha x^{*} e^{-i\omega _{0} \tau _{0}}}, \\& q_{2}^{*}= \frac{(i\omega _{0}-u) (-\rho +\beta x^{*}e^{i\omega _{0} \tau _{0}} )-k\alpha x^{*}e^{i\omega _{0}\tau _{0}}}{(i\omega _{0}-u) [i\omega _{0}-(a+\rho )+\beta x^{*}e^{i\omega _{0} \tau _{0}} ]+k \alpha x^{*}e^{i\omega _{0}\tau _{0}}}, \\& h_{3}^{*}= \frac{\alpha x^{*} e^{i\omega _{0}\tau _{0}}-\alpha x^{*} e ^{i\omega _{0}\tau _{0}}h_{2}^{*}}{i\omega _{0}-u}, \end{aligned}$$

and

$$ \bigl\langle h^{*}(s),h(\theta )\bigr\rangle =1, \quad\quad \bigl\langle h^{*}(s),\bar{h}( \theta )\bigr\rangle =0, $$

where

$$\begin{aligned}& D= \bigl\{ 1+h_{2}\bar{h_{2}^{*}}+h_{3} \bar{h_{3}^{*}}+\tau _{0}A \bigr\} ^{-1}, \\& \begin{aligned} H&= -\bigl(\beta y^{*}+\alpha v^{*} \bigr)e^{-i\omega _{0}\tau _{0}}-\beta x^{*} e ^{-i\omega _{0}\tau _{0}}h_{2}- \alpha x^{*} e^{-i\omega _{0}\tau _{0}}h _{3}+\bar{h_{2}^{*}} \bigl(\bigl(\beta y^{*}+\alpha v^{*}\bigr)e^{-i\omega _{0}\tau _{0}} \\ &\quad{} +\beta x^{*} e^{-i\omega _{0}\tau _{0}}h_{2}+\alpha x^{*} e^{-i\omega _{0}\tau _{0}}h_{3} \bigr). \end{aligned} \end{aligned}$$

Using the same notations as in [47], we can obtain

$$\begin{aligned}& \begin{aligned} g_{20}&= 2D\bigl(1,\bar{h_{2}^{*}}, \bar{h_{3}^{*}}\bigr) \begin{pmatrix} -(\beta h_{2}+\alpha h_{3})e^{-2i\omega _{0}\tau _{0}} \\ (\beta h_{2}+\alpha h_{3})e^{-2i\omega _{0}\tau _{0}} \\ 0 \end{pmatrix} \\ &= 2D \bigl[-(\beta h_{2}+\alpha h_{3})e^{-2i\omega _{0}\tau _{0}}+ \bar{h _{2}^{*}}(\beta h_{2}+\alpha h_{3})e^{-2i\omega _{0}\tau _{0}} \bigr], \end{aligned} \\& \begin{aligned} g_{11}&= D\bigl(1,\bar{h_{2}^{*}}, \bar{h_{3}^{*}}\bigr) \begin{pmatrix} -\beta (\bar{h_{2}}+h_{2})-\alpha (\bar{h_{3}}+h_{3}) \\ \beta (\bar{h_{2}}+h_{2})+\alpha (\bar{h_{3}}+h_{3}) \\ 0 \end{pmatrix} \\ &= D \bigl[\beta (\bar{h_{2}}+h_{2})+\alpha ( \bar{h_{3}}+h_{3}) \bigr] \bigl(\bar{h_{2}^{*}}-1 \bigr), \end{aligned} \\& \begin{aligned} g_{02}&= 2D\bigl(1,\bar{h_{2}^{*}}, \bar{h_{3}^{*}}\bigr) \begin{pmatrix} -(\beta \bar{h_{2}}+\alpha \bar{h_{3}})e^{2i\omega _{0}\tau _{0}} \\ (\beta \bar{h_{2}}+\alpha \bar{h_{3}})e^{2i\omega _{0}\tau _{0}} \\ 0 \end{pmatrix} \\ &= 2D(\beta \bar{h_{2}}+\alpha \bar{h_{3}})e^{2i\omega _{0}\tau _{0}} \bigl(\bar{h _{2}^{*}}-1\bigr), \end{aligned} \\& \begin{aligned} g_{21}&= 2D\bigl(1,\bar{h_{2}^{*}}, \bar{h_{3}^{*}}\bigr) \begin{pmatrix} B \\ C \\ 0 \end{pmatrix}, \end{aligned} \end{aligned}$$

where

$$\begin{aligned}& \begin{aligned} B&= -\beta \biggl[w_{11}^{(2)}(-\tau _{0})e^{-i\omega _{0}\tau _{0}}+ \frac{1}{2}w_{20}^{(2)}(- \tau _{0})e^{-i\omega _{0}\tau _{0}} + \frac{1}{2}w_{20}^{(1)}(- \tau _{0})\bar{h_{2}}e^{-i\omega _{0}\tau _{0}} \\ &\quad{}+w _{11}^{(1)}(- \tau _{0})h_{2}e^{-i\omega _{0}\tau _{0}} \biggr] \\ &\quad{} -\alpha \biggl[w_{11}^{(3)}(-\tau _{0})e^{-i\omega _{0}\tau _{0}}+ \frac{1}{2}w_{20}^{(3)}(- \tau _{0})e^{-i\omega _{0}\tau _{0}}+ \frac{1}{2}w_{20}^{(1)}(- \tau _{0})\bar{h_{3}}e^{-i\omega _{0}\tau _{0}} \\ &\quad{}+w _{11}^{(1)}(- \tau _{0})h_{3}e^{-i\omega _{0}\tau _{0}} \biggr], \end{aligned} \\& \begin{aligned} C&= \beta \biggl[w_{11}^{(2)}(-\tau _{0})e^{-i\omega _{0}\tau _{0}}+ \frac{1}{2}w_{20}^{(2)}(- \tau _{0})e^{-i\omega _{0}\tau _{0}}+ \frac{1}{2}w_{20}^{(1)}(- \tau _{0})\bar{h_{2}}e^{-i\omega _{0}\tau _{0}}+w _{11}^{(1)}(- \tau _{0})h_{2}e^{-i\omega _{0}\tau _{0}} \biggr] \\ &\quad{} +\alpha \biggl[w_{11}^{(3)}(-\tau _{0})e^{-i\omega _{0}\tau _{0}} + \frac{1}{2}w_{20}^{(3)}(- \tau _{0})e^{-i\omega _{0}\tau _{0}}+ \frac{1}{2}w_{20}^{(1)}(- \tau _{0})\bar{h_{3}}e^{-i\omega _{0}\tau _{0}} \\ &\quad{}+w _{11}^{(1)}(- \tau _{0})h_{3}e^{-i\omega _{0}\tau _{0}} \biggr], \end{aligned} \\& w_{20}(\theta )= \frac{ig_{20}}{\omega _{0}}h(0)e^{i\omega _{0}\theta }+ \frac{i\bar{g _{20}}}{3\omega _{0}}\bar{h}(0)e^{-i\omega _{0}\theta }+E_{1}e^{2i\omega _{0}\theta }, \\& w_{11}(\theta )= \frac{-ig_{11}}{\omega _{0}}h(0)e^{i\omega _{0}\theta }+ \frac{i\bar{g_{11}}}{\omega _{0}}\bar{h}(0)e^{-i\omega _{0}\theta }+E _{2}, \end{aligned}$$

with

$$\begin{aligned} E_{1}&= \begin{pmatrix} 2i\omega _{0}+d+(\beta y^{*}+\alpha v^{*})e^{-2i\omega _{0}\tau _{0}} & \beta x^{*}e^{-2i\omega _{0}\tau _{0}}-\rho & dx^{*}e^{-2i\omega _{0}\tau _{0}} \\ -(\beta y^{*}+\alpha v^{*})e^{-2i\omega _{0}\tau _{0}} & 2i\omega _{0}+a+ \rho -\beta x^{*}e^{-2i\omega _{0}\tau _{0}} & \alpha x^{*}e^{-2i\omega _{0}\tau _{0}} \\ 0 & -k & 2i\omega _{0}+u \end{pmatrix}^{-1} \\ &\quad{} \times \begin{pmatrix} -(\beta h_{2}+\alpha h_{3})e^{-2i\omega _{0}\tau _{0}} \\ (\beta h_{2}+\alpha h_{3})e^{-2i\omega _{0}\tau _{0}} \\ 0 \end{pmatrix}, \end{aligned}$$

and

$$ E_{2}= \begin{pmatrix} -d-(\beta y^{*}+\alpha v^{*}) & \rho -\beta x^{*} & -\alpha x^{*} \\ \beta y^{*}+\alpha v^{*} & \beta x^{*}-(a+\rho ) & \alpha x^{*} \\ 0 & k & -u \end{pmatrix}^{-1} \begin{pmatrix} \beta (\bar{h_{2}}+h_{2})+\alpha (\bar{h_{3}}+h_{3}) \\ -\beta (\bar{h_{2}}+h_{2})-\alpha (\bar{h_{3}}+h_{3}) \\ 0 \end{pmatrix}. $$

By substituting $E_{1}$ and $E_{2}$ into $W_{20}(\theta )$ and $W_{11}(\theta )$, respectively, $g_{21}$ can be expressed by the parameters. Then each $g_{ij}$ can be determined by the parameters. Therefore we get the following expression:

$$\begin{aligned}& C_{1}(0)= \frac{i}{2\omega _{0}}\biggl(g_{20}g_{11}-2 \vert g_{11} \vert ^{2}- \frac{1}{3} \vert g_{02} \vert ^{2}\biggr)+\frac{g_{21}}{2}, \quad\quad \mu _{2}=-\frac{ \operatorname{Re}\{C_{1}(0)\}}{\operatorname{Re}\lambda ^{\prime }(\tau _{0})}, \\& T_{2}= -\frac{\operatorname{Im}\{C_{1}(0)\}+\mu _{2}\operatorname{Im} \lambda ^{\prime }(\tau _{0})}{\omega _{0}}, \quad\quad \beta _{2}=2 \operatorname{Re}\bigl\{ C_{1}(0)\bigr\} . \end{aligned}$$

Thus, we have from [47] the following theorem.

### Theorem 4.1

(i) $\mu _{2}$*determines the direction of Hopf bifurcation*. *If* $\mu _{2}>0$ (<0), *then Hopf bifurcation is supercritical* (*subcritical*).
(ii) $\beta _{2}$*determines the stability of the bifurcated periodic solutions*. *If* $\beta _{2}<0$ (>0), *then the bifurcated periodic solutions are orbitally stable* (*unstable*).
(iii) $T_{2}$*determines the period of the bifurcated periodic solutions*. *If* $T_{2}>0$ (<0), *then the period increases* (*decreases*).

## Conclusion and numerical simulations

In this paper, we have mainly considered the effect of time delay on the dynamics of a virus model with two different transmission methods and treatments. Our results show that the introduction of time delay has a significant effect on the dynamics of the system. However, it can be seen from [1] that when there is no time delay in system (3), the positive equilibrium $E^{*}$ of system (3) is asymptotically stable if it exists. The appearance of the time delay causes the positive equilibrium of the model (4) to be inverted from stable to unstable, and a periodic solution of small amplitude is generated near the positive equilibrium $E^{*}$. Biologically, the number of healthy, infected, and free viruses exhibits periodic changes.

**Figure 1:**
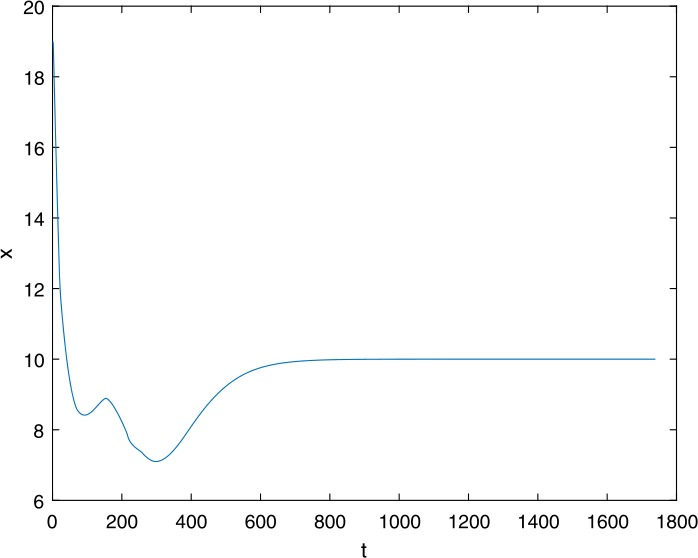
Time series for $x(t)$ with the initial value $(19, 40, 30581)$, where $\mathscr{R}=0.514971<1$

**Figure 2:**
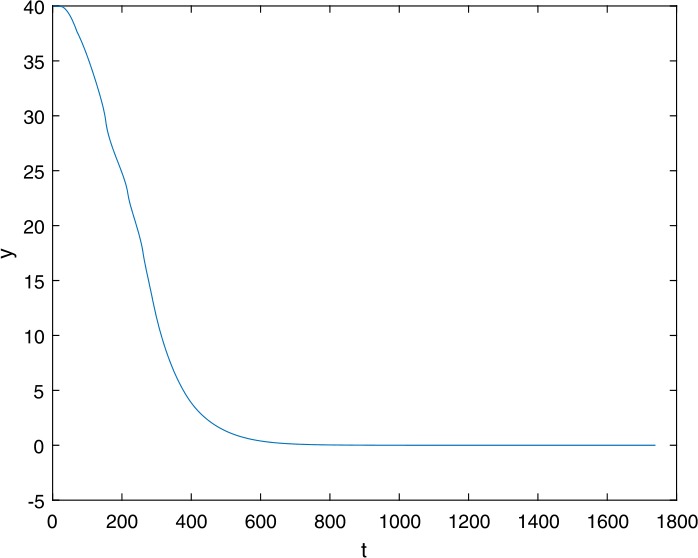
Time series for $y(t)$ with the initial value $(19, 40, 30581)$, where $\mathscr{R}=0.514971<1$

**Figure 3:**
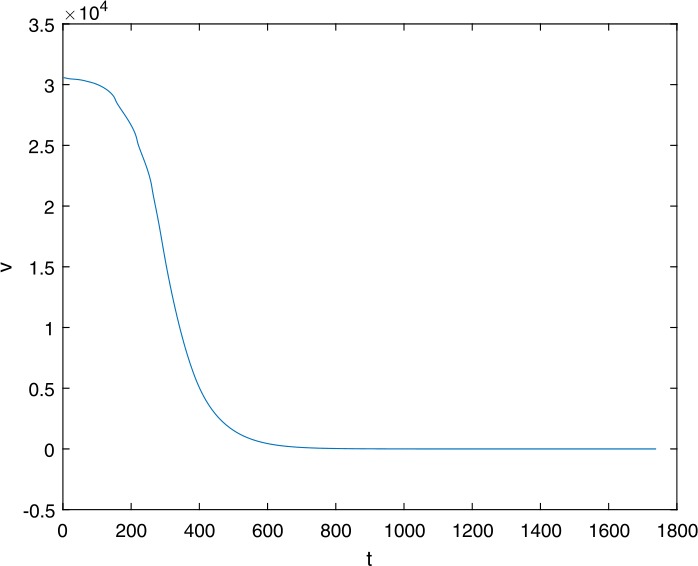
Time series for $v(t)$ with the initial value $(19, 40, 30581)$, where $\mathscr{R}=0.514971<1$

**Figure 4:**
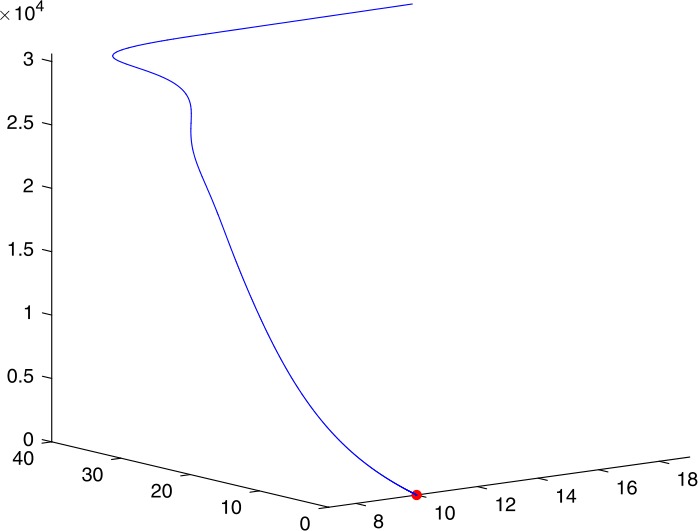
3D phase for $x(t)$, $y(t)$, and $v(t)$ with the initial value $(19, 40, 30581)$, where $\mathscr{R}=0.514971<1$

Next, we take some numerical simulations to validate our main results. We set the basic parameters as follows [48]: $d= 0.2$, $\beta = 0.000024$, $\alpha = 0.000024$, $\rho =0.2$, $a=0.15$, $k=150$, $u=0.2$. Firstly, we set $\pi = 2 $, direct calculations with Maple 14 show that $\mathscr{R}=0.514971<1 $ and $E_{0}=(10, 0, 0)$. By Theorem 3.1 the virus-free equilibrium $E_{0}$ of the system is stable (see Figs. 1–4). Next, we change *π* to 10, direct calculations show that $\mathscr{R}=2.574857>1$, then system (4) has two equilibria, i.e., the virus-free equilibrium $E_{0}=(50, 0, 0)$ and the virus equilibrium $E^{*}=(19.4186,40.7753,30581.4470)$. It is easy to see that

$$\begin{aligned}& b_{0} = 0.0140, \\& c_{0} =0.008048, \\& G(0) =d_{0}=b_{0}^{2}-c_{0}^{2}=0.000131>0, \\& \Delta =0.148921>0, \\& z_{1}^{*} =0.240948, \\& G\bigl(z_{1}^{*}\bigr) =-0.008284< 0, \end{aligned}$$

then equation (12) has two positive roots $z_{1}=0.0089$ and $z_{2}=0.3680$, and equation (11) has two positive roots $\varpi _{1}=0.09441603258$ and $\varpi _{2}=0.6066529567$. Therefore we have the Hopf bifurcation value $\tau _{2}^{0} = 3.828340005$.

If we set $\tau = 3.8<3.828340005$, by Theorem 3.2, the virus equilibrium $E^{*}$ is asymptotically stable (see Figs. 5–8). If we set $\tau = 3.9>3.828340005$, by Theorem 3.2, the virus equilibrium $E^{*}$ is unstable and periodic oscillations occur (see Figs. 9–16 with different initial values).

**Figure 5:**
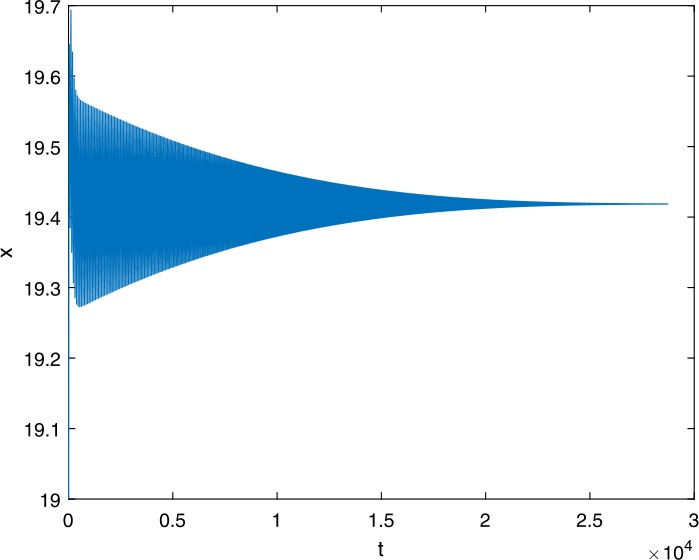
Time series for $x(t)$ with the initial value $(19, 40, 30581)$, where $\mathscr{R}=2.574857>1$

**Figure 6:**
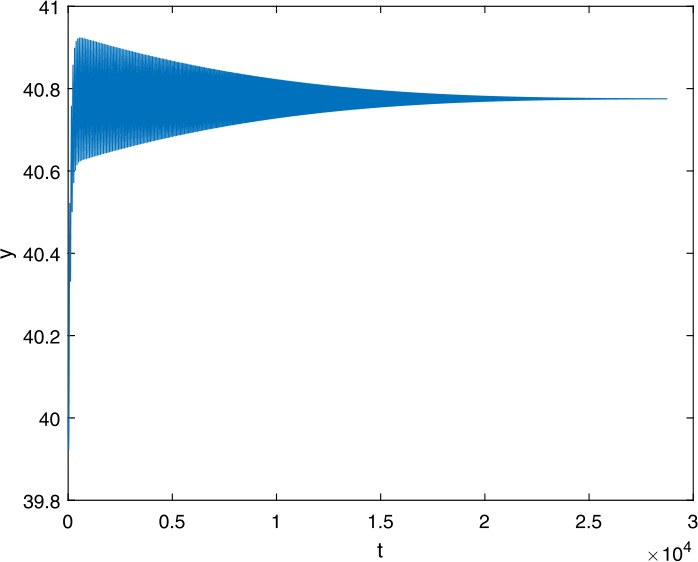
Time series for $y(t)$ with the initial value $(19, 40, 30581)$, where $\mathscr{R}=2.574857>1$

**Figure 7:**
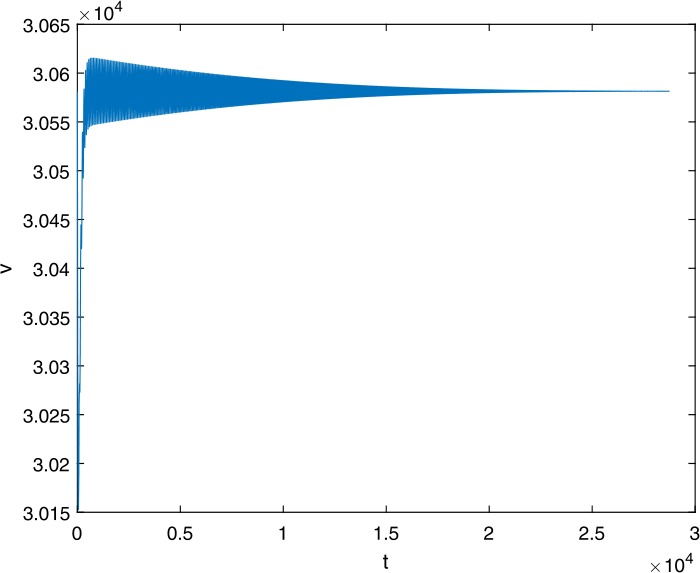
Time series for $v(t)$ with the initial value $(19, 40, 30581)$, where $\mathscr{R}=2.574857>1$

**Figure 8:**
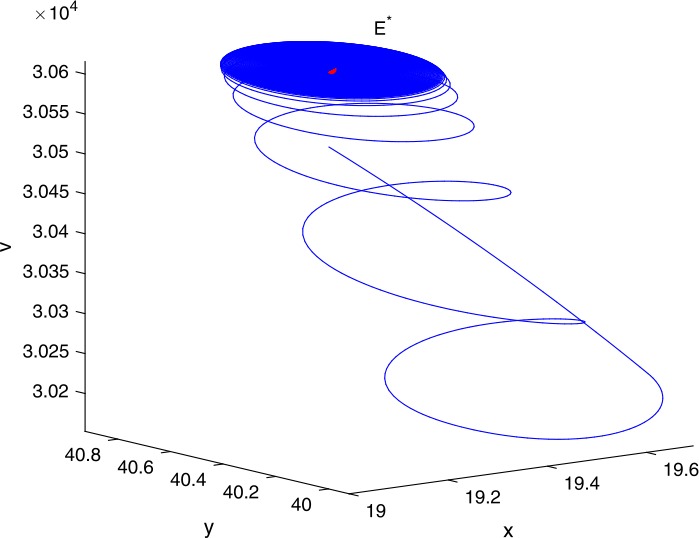
3D phase for $x(t)$, $y(t)$, and $v(t)$ with the initial value $(19, 40, 30581)$, where $\mathscr{R}=2.574857>1$

**Figure 9:**
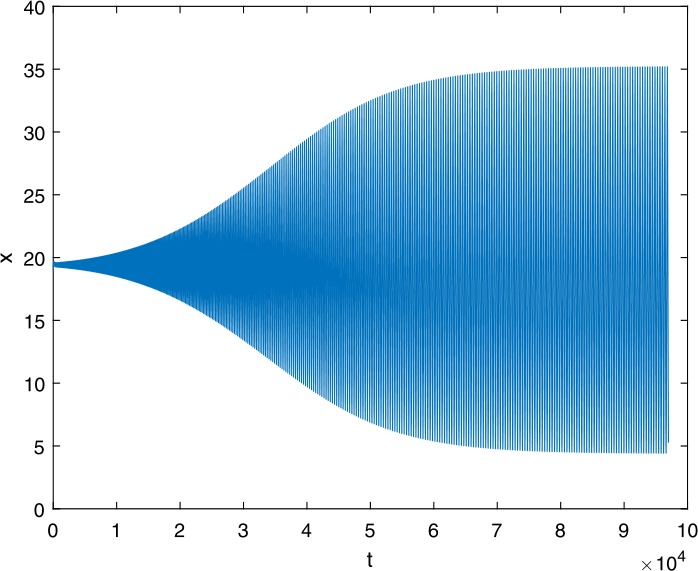
Time series for $x(t)$ with the initial value $(19, 40, 30581)$, where $\mathscr{R}=2.574857>1$, $\tau = 3.8< \tau _{2}^{0}$

**Figure 10:**
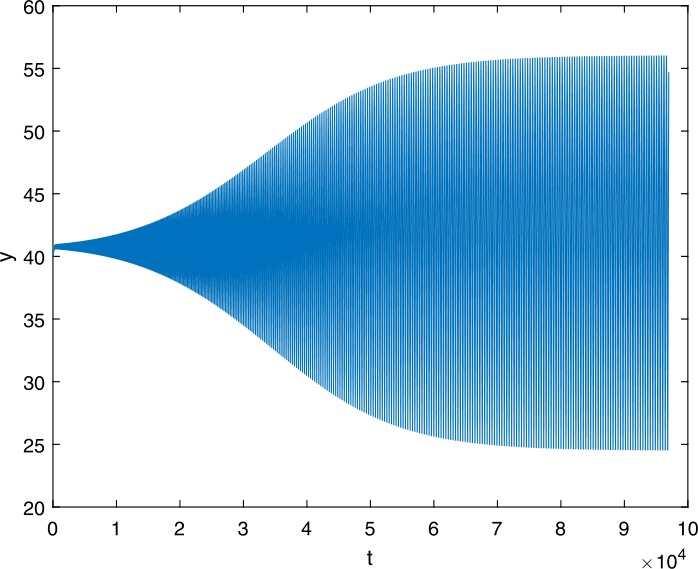
Time series for $y(t)$ with the initial value $(19, 40, 30581)$, where $\mathscr{R}=2.574857>1$, $\tau = 3.8< \tau _{2}^{0}$

**Figure 11:**
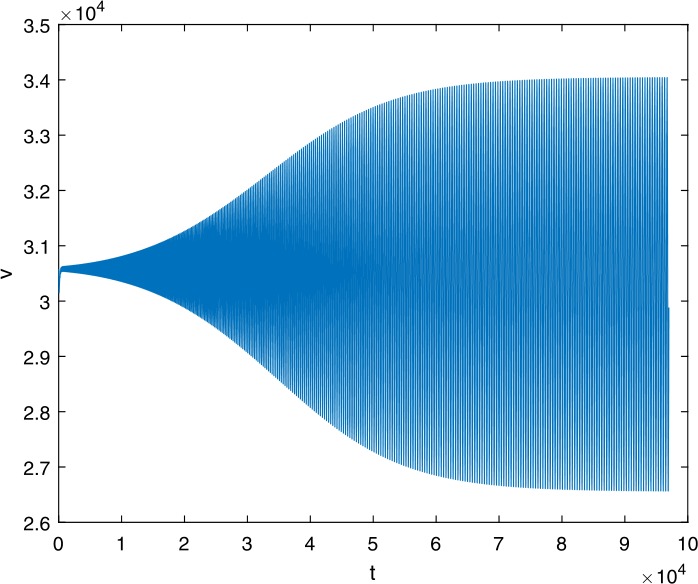
Time series for $v(t)$ with the initial value $(19, 40, 30581)$, where $\mathscr{R}=2.574857>1$, $\tau = 3.8< \tau _{2}^{0}$

**Figure 12:**
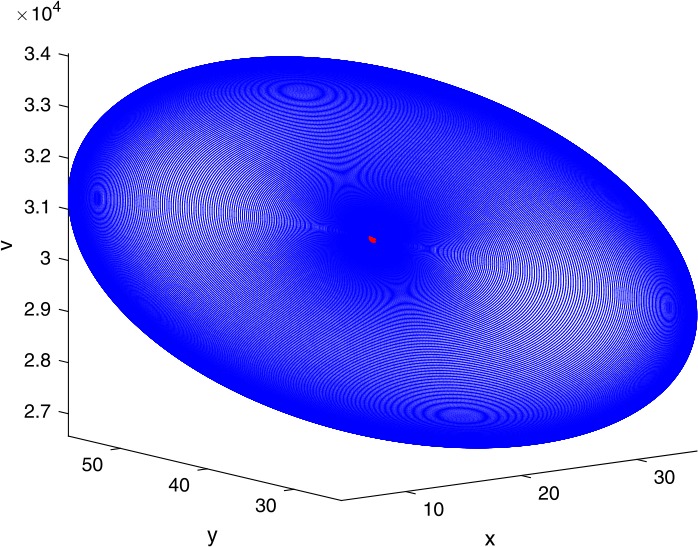
3D phase for $x(t)$, $y(t)$, and $v(t)$ with the initial value $(19, 40, 30581)$, where $\mathscr{R}=2.574857>1$, $\tau = 3.8<\tau _{2}^{0}$

**Figure 13:**
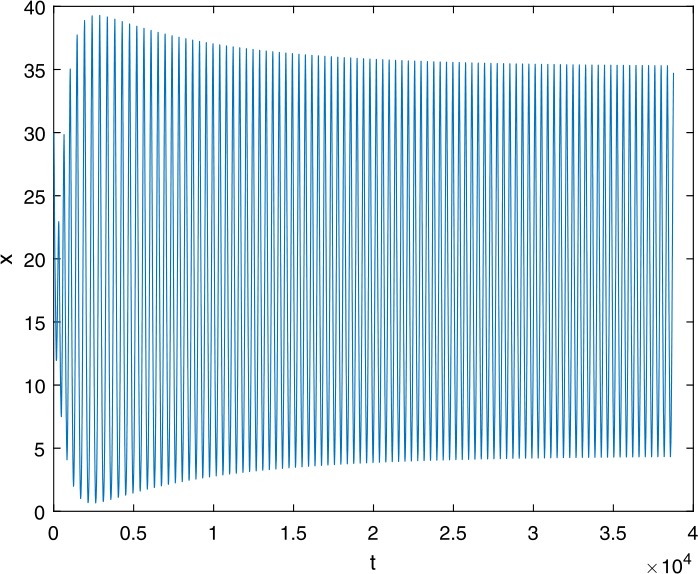
Time series for $x(t)$ with the initial value $(30, 75, 29000)$, where $\mathscr{R}=2.574857>1$, $\tau = 3.9> \tau _{2}^{0}$

**Figure 14:**
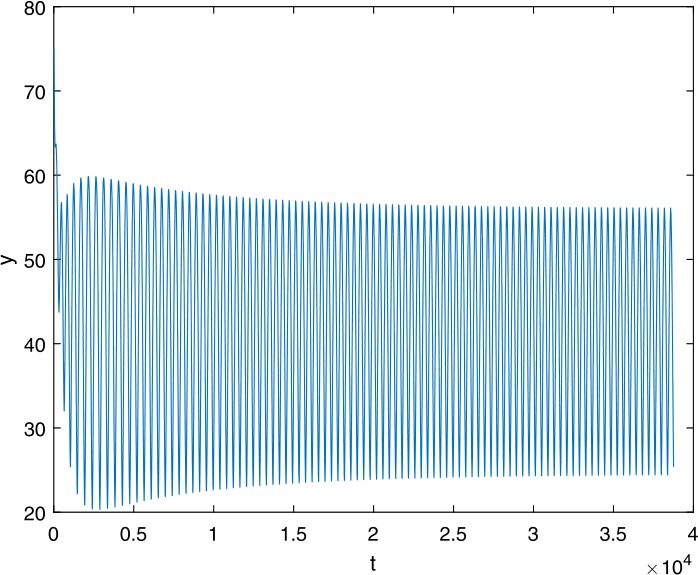
Time series for $y(t)$ with the initial value $(30, 75, 29000)$, where $\mathscr{R}=2.574857>1$, $\tau = 3.9> \tau _{2}^{0}$

**Figure 15:**
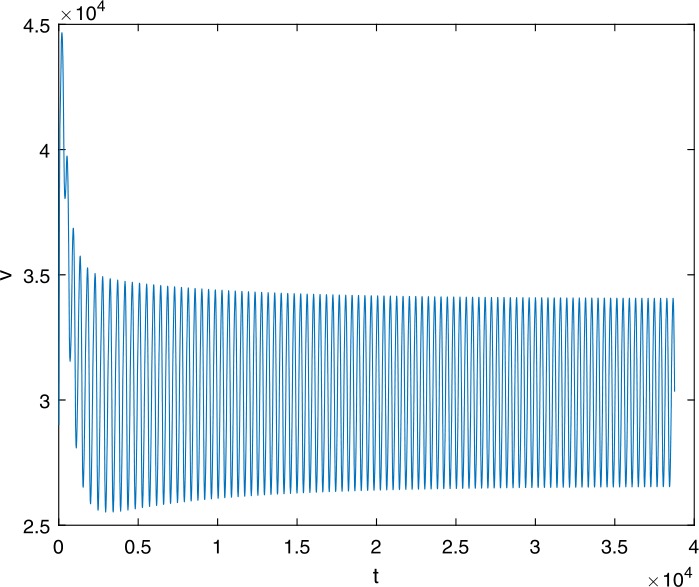
Time series for $v(t)$ with the initial value $(30, 75, 29000)$, where $\mathscr{R}=2.574857>1$, $\tau = 3.9> \tau _{2}^{0}$

**Figure 16:**
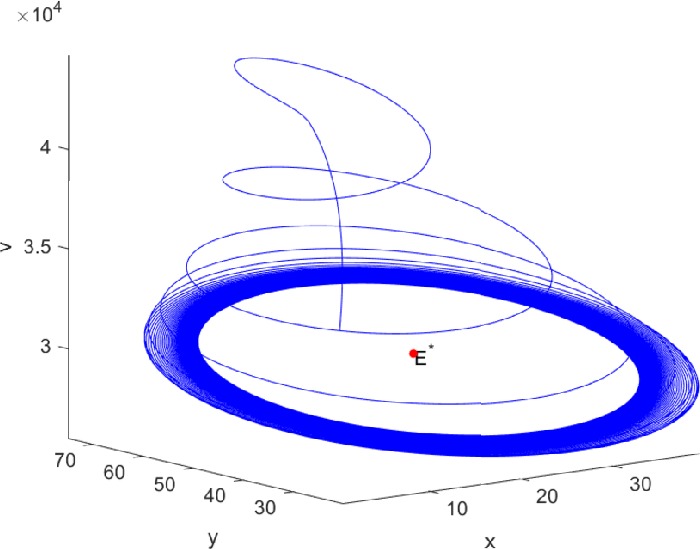
3D phase for $x(t)$, $y(t)$, and $v(t)$ with the initial value $(30, 75, 29000)$, where $\mathscr{R}=2.574857>1$, $\tau = 3.9>\tau _{2}^{0}$

## References

[1] Zhang T., Meng X., Zhang T. (2015). Global dynamics of a virus dynamical model with cell-to-cell transmission and cure rate. Comput. Math. Methods Med..

[2] WHO: World health organization report: research for universal health coverage. Technical report, World Health Organization, (2013). http://www.who.int/whr/en/

[3] Zhang W., Meng X., Dong Y. (2019). Periodic solution and ergodic stationary distribution of stochastic SIRI epidemic systems with nonlinear perturbations. J. Syst. Sci. Complex..

[4] Division of STD Prevention: Prevention of Genital HPV Infection and Sequelae: Report of an External Consultants’ Meeting. Department of Health and Human Services, Atlanta: Centers for Disease Control (1999)

[5] Winer R.L., Hughes J.P., Feng Q., O’Reilly S., Kiviat N.B., Holmes K.K., Koutsky L.A. (2006). Condom use and the risk of genital human papillomavirus infection in young women. N. Engl. J. Med..

[6] Daling J.R., Madeleine M.M., Johnson L.G., Schwartz S.M., Shera K.A., Wurscher M.A., Carter J.J., Porter P.L., Galloway D.A., Mcdougall J.K. (2004). Human papillomavirus, smoking, and sexual practices in the etiology of anal cancer. Cancer.

[7] Parkin M., Bray F. (2006). The burden of HPV-related cancers. Vaccine.

[8] Chaturvedi A.K., Engels E.A., Pfeiffer R.M., Hernandez B.Y., Xiao W., Kim E., Jiang B., Goodman M.T., Sibugsaber M., Cozen W. (2011). Human papillomavirus and rising oropharyngeal cancer incidence in the United States. J. Clin. Oncol..

[9] Cui J., Zhang Y., Feng Z., Guo S., Zhang Y. (2019). Influence of asymptomatic infections for the effectiveness of facemasks during pandemic influenza. Math. Biosci. Eng..

[10] Feng T., Qiu Z. (2019). Global analysis of a stochastic TB model with vaccination and treatment. Discrete Contin. Dyn. Syst., Ser. B.

[11] Zhao W., Liu J., Chi M., Bian F. (2019). Dynamics analysis of stochastic epidemic models with standard incidence. Adv. Differ. Equ..

[12] Feng T., Qiu Z., Meng X. (2019). Analysis of a stochastic recovery-relapse epidemic model with periodic parameters and media coverage. J. Appl. Anal. Comput..

[13] Zhou X., Song X., Shi X. (2008). A differential equation model of HIV infection of CD4^+^ T-cells with cure rate. J. Math. Anal. Appl..

[14] Li J., Song X., Gao F. (2012). Global stability of a viral infection model with two delays and two types of target cells. J. Appl. Anal. Comput..

[15] Xu R. (2011). Global stability of an HIV-1 infection model with saturation infection and intracellular delay. J. Math. Anal. Appl..

[16] Shu H., Wang L. (2012). Role of cd4^+^ t-cell proliferation in HIV infection under antiretroviral therapy. J. Math. Anal. Appl..

[17] Guidotti L.G., Rochford R., Chung J., Shapiro M., Purcell R., Chisari F.V. (1999). Viral clearance without destruction of infected cells during acute HBV infection. Science.

[18] Wang K., Fan A., Torres A. (2010). Global properties of an improved hepatitis B virus model. Nonlinear Anal., Real World Appl..

[19] Dahari H., Shudo E., Ribeiro R.M., Perelson A.S. (2009). Modeling complex decay profiles of hepatitis B virus during antiviral therapy. Hepatology.

[20] Qian H., Ning P., Wei D. (2013). Global stability for a dynamic model of hepatitis B with antivirus treatment. J. Appl. Anal. Comput..

[21] Feng T., Qiu Z., Meng X., Rong L. (2019). Analysis of a stochastic HIV-1 infection model with degenerate diffusion. Appl. Math. Comput..

[22] Cui J., Ying C., Guo S., Zhang Y., Sun L., Zhang M., He J., Song T. (2019). Influence of media intervention on AIDS transmission in MSM groups. Math. Biosci. Eng..

[23] Ji Y., Ma W., Song K. (2018). Modeling inhibitory effect on the growth of uninfected T cells caused by infected T cells: stability and Hopf bifurcation. Comput. Math. Methods Med..

[24] Guo S., Ma W. (2016). Global behavior of delay differential equations model of HIV infection with apoptosis. Discrete Contin. Dyn. Syst., Ser. B.

[25] Song Y., Miao A., Zhang T., Wang X., Liu J. (2018). Extinction and persistence of a stochastic SIRS epidemic model with saturated incidence rate and transfer from infectious to susceptible. Adv. Differ. Equ..

[26] Gao N., Song Y., Wang X., Liu J. (2019). Dynamics of a stochastic SIS epidemic model with nonlinear incidence rates. Adv. Differ. Equ..

[27] Miao A., Zhang T., Zhang J., Wang C. (2018). Dynamics of a stochastic SIR model with both horizontal and vertical transmission. J. Appl. Anal. Comput..

[28] Fan X., Song Y., Zhao W. (2018). Modeling cell-to-cell spread of HIV-1 with nonlocal infection. Complexity.

[29] Perelson A.S., Nelson P.W. (1999). Mathematical analysis of HIV-1 dynamics in vivo. SIAM Rev..

[30] Perelson A.S., Neumann A.U., Markowitz M., Leonard J.M., Ho D.D. (1996). HIV-1 dynamics in vivo: virion clearance rate, infected cell life-span, and viral generation time. Science.

[31] Nelson P.W., Perelson A.S. (2002). Mathematical analysis of delay differential equation models of HIV-1 infection. Math. Biosci..

[32] Mcdonald D., Hope T.J. (2003). Recruitment of HIV and its receptors to dendritic cell–T cell junctions. Science.

[33] Sattentau Q. (2008). Avoiding the void: cell–to–cell spread of human viruses. Nat. Rev. Microbiol..

[34] Phillips D.M. (1994). The role of cell–to–cell transmission in HIV infection. AIDS.

[35] Sato H., Orensteint J., Dimitrov D., Martin M. (1992). Cell-to-cell spread of HIV-1 occurs within minutes and may not involve the participation of virus particles. Virology.

[36] Jolly C. (2011). Cell–to–cell transmission of retroviruses: innate immunity and interferon-induced restriction factors. Virology.

[37] Bangham C.R. (2003). The immune control and cell–to–cell spread of human T–lymphotropic virus type 1. J. Gen. Virol..

[38] Krantic S., Gimenez C., Rabourdincombe C. (1995). Cell–to–cell contact via measles virus haemagglutinin-CD46 interaction triggers CD46 downregulation. J. Gen. Virol..

[39] Wild T.F., Malvoisin E., Buckland R. (1991). Measles virus: both the haemagglutinin and fusion glycoproteins are required for fusion. J. Gen. Virol..

[40] Spouge J.L., Shrager R.I., Dimitrov D.S. (1996). HIV-1 infection kinetics in tissue cultures. Math. Biosci..

[41] Herz A.V., Bonhoeffer S., Anderson R.M., May R.M., Nowak M.A. (1996). Viral dynamics in vivo: limitations on estimates of intracellular delay and virus decay. Proc. Natl. Acad. Sci..

[42] Culshaw R.V., Ruan S., Webb G. (2003). A mathematical model of cell-to-cell spread of HIV-1 that includes a time delay. J. Math. Biol..

[43] Lai X., Zou X. (2015). Modeling cell-to-cell spread of HIV-1 with logistic target cell growth. J. Math. Anal. Appl..

[44] Routh E.J., Clifford W.K., Sturm C., Bocher M. (1975). Stability of Motion.

[45] Ruan S., Wei J. (2001). On the zeros of a third degree exponential polynomial with applications to a delayed model for the control of testosterone secretion. IMA J. Math. Appl. Med. Biol..

[46] Hassard B.D., Kazarinoff N.D., Wan Y.H. (1981). Theory and Applications of Hopf Bifurcation.

[47] Hale J., Lunel M. (1993). Introduction to Functional Differential Equations.

[48] Zhuang K., Zhu H. (2013). Stability and bifurcation analysis for an improved HIV model with time delay and cure rate. WSEAS Trans. Math..

